# Editorial: Radiation therapy and organ preservation: controversies and emerging evidence

**DOI:** 10.3389/fonc.2026.1918886

**Published:** 2026-07-20

**Authors:** Sangjune Laurence Lee, Hamid Raziee, Ezra Hahn, Rahul Krishnatry, Srinivas Raman

**Affiliations:** 1 University of Calgary, Calgary, AB, Canada; 2 Arthur Child Cancer Centre, Calgary, Canada; 3 British Columbia (BC) Cancer Kelowna, Kelowna, BC, Canada; 4 Princess Margaret Hospital Cancer Center, Toronto, ON, Canada; 5 Tata Memorial Hospital, Mumbai, Maharashtra, India; 6 British Columbia (BC) Cancer, Vancouver, BC, Canada

**Keywords:** benign, organ preservation, radiation therapy (radiotherapy), spatially fractionated radiation therapy, therapeutic ratio

The paradigm of organ preservation strategies in cancer management has seen significant advancements, offering patients the potential for improved quality of life without compromising survival outcomes (1–4). Advances in imaging, treatment planning, and delivery have positioned radiation therapy (RT) as a primary catalyst and cornerstone for many organ preservation strategies. The central challenge is the optimization of the therapeutic ratio: maximizing dose to the target while minimizing the “collateral damage” to surrounding healthy tissues (5). There is a need for further investigations and a multidisciplinary perspective on calibrating treatment intensity while balancing the goals of cancer control, organ function, and quality of life.

This Research Topic brings together a diverse collection of 13 articles—ranging from systematic reviews and meta-analyses to novel case reports—that examine the clinical implementation, technological hurdles, and emerging biological evidence for preserving vital organs without compromising oncologic control.

Minimizing radiation toxicity is a central theme of this special edition. Xie at al. describe a dosimetric analysis of 20 patients undergoing breast radiotherapy and recommend the delineation of cardiac substructures for cardiac sparing approaches to minimize late cardiovascular complications while balancing coverage of the IMN elective nodes. Yu et al. provide a review of the use of a dose-function histogram to supplant the traditional dose-volume histogram to avoid radiation-induced lung injury. Kemin and Rutie review progress in the prevention and treatment of radiation-induced skin damage and provide intriguing data on the use of traditional Chinese medicine in addition to Western medicine. Liu et al. provide a comprehensive review of hippocampal sparing in whole-brain radiotherapy for brain metastases to limit cognitive dysfunction.

The role of radiotherapy in the management of cancer has evolved and is also being explored in the management of “benign” conditions with major impacts on quality of life. Pu et al. systematically review and provide a meta-analysis on the use of total neoadjuvant therapy based short course radiotherapy and compare it to the traditional long course chemoradiotherapy for locally advanced rectal cancer and show that short course radiotherapy based total neoadjuvant therapy has higher pathologic response, convenience and cost savings. Kim et al. present a case of an extensive, recurrent pelvic venous malformation treated with RT and resulting in durable disease control while preserving ovarian function. Zong et al. describe a patient with a disfiguring hypertrophic scar that responds to superficial RT by reshaping the wound repair process.

Improved and innovative RT delivery techniques can further improve the tumoricidal effects while minimizing normal tissue toxicity. McGarrigle et al. systematically review the use of different geometrical and dosimetric parameters in microbeam and minibeam RT which are specialized RT techniques under the umbrella of Spatially Fractionated RT (SFRT) and identify critical geometric and dosimetric parameters for sparing healthy tissue. Wu et al. describe the dosimetry of half-field-based VMAT for patients with left breast cancer with resultant enhanced protection of OARs. Liu et al. propose and validate a novel weighted-Dose-Accuracy-Product (DAPw) index for balancing RT setup accuracy and imaging radiation dose.

The delivery of effective RT also depends on novel organ sparing innovations, systemic therapy sequencing and RT access infrastructure. Woo et al. present their institution’s experience of injecting a hydrogel spacer to reduce rectal toxicity during prostate radiotherapy and show that rates of rectal wall infiltration with the gel are uncommon and not associated with increased complications. Yang et al.review strategies to mitigate the occurrence of radionecrosis patients undergoing RT and BRAF inhibitors for lung adenocarcinoma brain metastases. Chen et al. review health-system interventions to meaningfully expand access to RT through equity-focused strategies.

While this special edition highlights recent developments in toxicity mitigation, the evolving role of RT for cancer and benign conditions, and novel RT delivery techniques, further studies are needed to explore new organ preservation strategies, patient preferences, risks associated with organ preservation, and available salvage options. Future trials comparing organ preservation to standard surgical management and studies to identify the barriers and challenges in adopting these strategies will be of ongoing interest.
